# Death of Dr. Primrose Blair, Late Physician of the Fleet, &c.; with the Appearances on Dissection

**Published:** 1819-07-01

**Authors:** 


### *Death of Dr. Primrose Blair, late Physician of the Fleet, &c.; with the Appearances on Dissection.*

By the EDITOR.

ABOUT the beginning of December, 1818, Dr. Blair called on me in the Albany, and introduced himself as a brother officer, who wished to consult me for a complaint which he had laboured under for some years, and which he considered as a disease of the liver. He had, at this time, an unhealthy look, with considerable anxiety and distress in his countenance, which was sallow, with blueish tints under the eyes, on the nose, and about the lips. His tongue was somewhat furred, his skin rather dry, his pulse about 80 and regular, his belly pendulous, his muscles very flabby, and, according to his own statement, he was greatly reduced in flesh and strength. He stated to me, that till within these two or three years, he had enjoyed good health; had visited various climates; livid freely but not intemperately, and drank from a pint to a bottle of wine in the day, with a glass or two of strong ale at night. He was now about 63. The complaint commenced with a failure of strength and appetite; a loss of breath on walking any distance, especially up a hill; an irregularity of bowels; depression of spirits, with irritability of temper; troubled and sedimentous urine; but particularly a pain in the *right side* of the thorax, from the edge of the scapula down to the floating ribs. This, together with an occasional pain in the right shoulder, induced him to attribute the symptoms above described, to a diseased liver.

On further inquiry, I found that he had a pretty constant dry hecking cough, unaccompanied by any expectoration, with occasional pains in both arms, down to the wrists, and in the left arm, down to the extremities of the ring and little finger. On examining the thorax and abdomen in the erect posture, I could only ascertain that *no enlargement* of the liver existed, and that the thorax sounded well in every part, excepting along, and on each side of the spine opposite to the heart. But from this circumstance nothing decisive could be inferred; since the chain of bones and mass of muscles in the above situation, render *percussion* of the thorax a very imperfect mean of diagnosis. In the supine posture, however, I found that *abdominal compression* caused a sense of distress in the chest, which evinced that all was not right in that quarter. I did not proceed farther in the investigation; nor did I ask any questions which might lead Dr. Blair to suppose that I suspected any organic affection of the heart, though my conviction of this was so strong, that I mentioned the circumstance to two medical friends at the time.

Observing his anxiety, I stated to him my belief that, the affection of his liver, though perhaps something more than *functional*, was, on the whole, of no very serious character; since enlargement there was none, and the function, though depraved, was by no means suspended. I therefore recommended him merely to pay attention to the state of his bowels; to take five grains of the pilula hydrargyri every third night; to bathe his feet and legs in the nitro-muriatic acid bath occasionally, and finally, to live abstemiously and quietly as possible. In about a month I saw him again, when he appeared much the same, but complained greatly of the pain in his right side, which induced him to be cupped there, but without any benefit accruing. He observed that his strength and flesh were certainly wasting away, and that he ate nothing with a relish. He had not yet tried the bath, and merely regulated his bowels by gentle aperients.

A few evenings afterwards I called at his lodgings, in St. Martin's Lane, and found him unusually cheerful. He said he had eaten a better dinner than for a considerable time past, and felt free from pain or uneasiness of any kind. He expressed a wish, if I had half an hour to spare, to relate to me an occurrence that happened to him in the West Indies; and drawing his chair a little nearer the fire, he commenced his narrative. He had not finished the first sentence, however, when he clapped his hand suddenly on the right and front part of his chest, and turned pale as death. I immediately felt for his pulse, but it was gone. I gave him some wine and water, which were standing on the table, and he began to revive, complaining of great pain and difficulty of breathing, while a cold perspiration covered his face, breast, and extremities. His friends, Messrs. Menzies and Pater-Noster were now sent for, by my direction, as I considered him in great danger. His restlessness and distress were now great. He could not sit a minute in any one posture, nor could he lie down at all. Occasionally the pulse would get up a little; but whenever it got to a degree of tolerable development, it would sink all at once, and syncope would supervene. This state continued for twelve hours, without much alteration, excepting a troublesome nausea and vomiting, together with a considerable inclination to stool, with which he was harrassed through the night. He expired at eight o'clock the next morning, attended by his friend Mr. Menzies to the last.

### POST MORTEM APPEARANCES.

On the following day Mr. Menzies, and myself, in the presence of Mr. Pater-Noster, Mr. Birckhead, Mr. Wilmore, and some other gentlemen, examined the body, when the following appearances were minuted. " A thick layer of yellow fat on the abdomen; cartilages of the ribs firmly ossified. On laying open the abdomen, several inches of the stomach, round the cardiac orifice, and along the lesser curvature, were black. The liver was not enlarged, but its structure diseased, and resembling granite, in its variegated texture. In each cavity of the chest about four or five pounds of blood, partly coagulated, partly in serum. The lungs perfectly sound. Pericardium sound. The heart completely covered with fat, in some places nearly an inch in thickness. The muscular structure, even of the left ventricle, was so wasted that it was, in some places, not thicker than a halfpenny. The cavities of the ventricles themselves not enlarged, nor the valvular apparatus apparently diseased. The arch of the aorta was dilated, and an aneurism of some size was situated a little below the arch. This aneurism had burst into the posterior mediastinum, which was filled with blood. From the posterior mediastinum the blood burst into both sides of the chest, until the extravasation amounted to the quantity abovementioned. The blood had even insinuated itself down under the crura of the diaphragm, and up between the peritoneal and muscular coats of the stomach, where the blackness appeared. No valvular disease nor specks of ossification about the heart or aorta.

*Remarks.* It is not easy to account for the obstinate pain in the right side of the chest, for certainly the extent of derangement of structure in the liver appeared inadequate to the production of this symptom. It was more likely one of those strange, anomalous, and distantly seated effects of the organic disease of the heart and of the enlarging aneurism of the aorta. The depression of spirits and irritability of temper are common to both cardiac and hepatic complaints. The irregularity of bowels, and the sedimentous state of the urine are fairly attributable to the hepatic affection.

since the secretion of this organ, in its degenerated structure, could not possibly be healthy. The pulse was, as usual, *res fallacissima.* It is, however, astonishing that the central organ of the circulation, in the embarrassed and morbid state in which we found it, could have so long distributed the blood, with apparent regularity, through all parts of the system! What share the free and full mode of living may have had in *causing* these disorganizations of the heart and liver, it is difficult to say; but there can be little doubt that life might have been considerably prolonged and rendered more comfortable by an early adoption of low regimen, aqueous drink, and corporeal quietude. In this respect the case offers an instructive lesson!

☞ It was in contemplation to have given a short biographical sketch of this amiable and excellent man, but the materials were too scanty, and the events of his life devoid of much interest. It was once his intention to start as a candidate for professional eminence in this metropolis, but a wise philosophy induced him to abandon that resolution, and——

"Along the cool sequester'd vale of life
To keep the noiseless tenor of his way." J. J.

*Albany, 20th June,* 1819.

